# Obesity, a Diet-Induced Inflammatory Disease

**DOI:** 10.3390/nu11102284

**Published:** 2019-09-24

**Authors:** Albert Lecube, Carolina López-Cano

**Affiliations:** 1 Endocrinology and Nutrition Department, Arnau de Vilanova University Hospital, Obesity, Diabetes and Metabolism (ODIM) Research Group, IRBLleida, University of Lleida, 25716 Lleida, Spain; karolopezc@gmail.com; 2 Centro de Investigación Biomédica en Red de Diabetes y Enfermedades Metabólicas Asociadas (CIBERDEM), Instituto de Salud Carlos III (ISCIII), 28029 Madrid, Spain

Obesity is a multifactorial and complex disease that continues to challenge patients and professional caregivers [1,2]. Its prevalence has increased dramatically in recent decades, which has boosted the risks of a variety of comorbid conditions such as type 2 diabetes, hypertension, dyslipidaemia and obstructive sleep apnoea. In fact, for a body mass index (BMI) above 25 kg/m^2^, each 5-point increment increases overall mortality by 30% [3].

The discovery that obesity produces a pathological inflammatory state has aroused interest in the underlying mechanisms that trigger the onset of this low-grade chronic inflammatory signalling, which is also called metabolic inflammation or meta-inflammation [4]. The review by Rogero et al. summarizes the scientific evidence that supports the claim that obesity should be considered an inflammatory disease a state that is not limited to white adipose tissue but also includes metabolically active organs, such as the liver, skeletal muscles, pancreas and the gastrointestinal tract [5].

Changes in the profile of polyunsaturated fatty acids that respond to modern eating patterns might be essential if we consider the role of these nutrients in the genesis of the inflammatory response. More interestingly, the inflammatory state promoted by diet also has consequences for the maintenance and progression of weight gain. Rodents that undergo induced obesity via high-fat diets are characterised by the development of inflammation in the hypothalamic territories, neuron injury and reactive gliosis [6]. Translational relevance for humans is highlighted when the increased gliosis signalling detected by magnetic resonance imaging in the midbasal hypothalamus is significantly increased in obese people compared with lean young individuals, and is positively correlated with BMI but not gender or age [6].

Obesity is a disease that has historically eluded effective medical therapy [7,8]. This fact, together with the discouraging results associated with dietary and behavioural treatments, have led to the progressive use of bariatric surgery [8]. However, 25%–30% of patients who underwent surgery have a weight response that is considered to be not adequate and/or do not resolve their comorbidities [9,10]. Therefore, better understanding the mechanisms that link inflammation with dietary content and quality is necessary. Furthermore, it is essential to lay the basis for continuing research for new drugs. Recent data suggest that a glucagon-like peptide-1 based therapy that delivers dexamethasone in the central nervous system and periphery improves hypothalamic and systemic inflammatory markers and diet-induced metabolic abnormalities [11]. Targeting the Toll-Like Receptor 4 (TLR4) might be another useful therapeutic strategy for the prevention and treatment of obesity and its associated inflammation state and comorbidities.

The TLR4 is a transmembrane protein that plays an essential role in promoting the expansion of obesity-induced inflammatory response by the innate immune system. TLR4 recognises saturated fatty acids, but not monounsaturated and polyunsaturated acids, linking a Western diet to the perpetuation of obesity comorbidities. This finding explains why the ingestion of a single meal rich in saturated fatty acids is positively correlated with postprandial inflammation and the expression of TLR4 in blood mononuclear cells [12,13]. In addition, continuous ingestion of saturated fatty acids induces intestinal dysbiosis, increases epithelial permeability of the small intestine and greater translocation of bacterial components (often called bacterial endotoxins or lipopolysaccharides, LPS) from the intestinal lumen to blood circulation and peripheral tissues [14]. If we consider that saturated fatty acids are also an essential component of bacterial endotoxins, this metabolic endotoxemia works in tandem with unhealthy diets to promote the perpetuation of the pro-inflammatory cascade via TLR4 activation.

We hope that health professionals who take care of patients with obesity will once again realise that one of the most effective therapeutic approach against this disease and its comorbidities is a healthy diet low in fatty acids.
